# Comparable performance of the NACC Uniform Data Set version 3 neuropsychological test battery in assessing longitudinal cognitive change for African American and White participants

**DOI:** 10.1002/alz.70889

**Published:** 2025-11-08

**Authors:** Kwun C. G. Chan, Hiroko H. Dodge, Mary Sano, Rhoda Au, Suzanne Craft, Allan I. Levey, Sandra Weintraub, Walter A. Kukull, Andrew J. Saykin, Lisa L. Barnes

**Affiliations:** ^1^ National Alzheimer's Coordinating Center University of Washington Seattle Washington USA; ^2^ Department of Biostatistics University of Washington Seattle Washington USA; ^3^ Department of Neurology Massachusetts General Hospital, Harvard Medical School Boston Massachusetts USA; ^4^ Department of Psychiatry Icahn School of Medicine at Mount Sinai New York New York USA; ^5^ Department of Anatomy & Neurobiology Boston University School of Medicine Boston Massachusetts USA; ^6^ Department of Neurology Emory University School of Medicine Atlanta Georgia USA; ^7^ Department of Gerontology and Geriatric Medicine Wake Forest School of Medicine Winston‐Salem North Carolina USA; ^8^ Mesulam Center for Cognitive Neurology and Alzheimer's Disease Center Northwestern University Feinberg School of Medicine Chicago Illinois USA; ^9^ Department of Neurology Washington University in St. Louis St. Louis Missouri USA; ^10^ Indiana Alzheimer's Disease Research Center Indiana University School of Medicine Indianapolis Indiana USA; ^11^ Department of Neurological Sciences and Rush Alzheimer's Disease Center Rush University Medical Center Chicago Illinois USA

**Keywords:** Alzheimer's Disease Research Center, cognition, dementia, National Alzheimer's Coordinating Center, neuropsychology

## Abstract

**INTRODUCTION:**

Alzheimer's Disease Research Centers have used the Uniform Data Set Version 3 (UDS3) neuropsychological battery since 2015, but whether it exhibits differential sensitivity to change across race is unknown. We examined whether the UDS3 cognitive battery was comparably sensitive to longitudinal change between African American and White participants.

**METHODS:**

Data were obtained from the National Alzheimer's Coordinating Center (NACC). Linear mixed‐effects models examined racial differences in baseline and longitudinal change in standardized test scores, controlling for age, sex, education, recruitment source, health factors, family history of dementia, and diagnostic groups defined by baseline and longitudinal changes in Clinical Dementia Rating (CDR) scores.

**RESULTS:**

Compared to White participants, African American participants had significantly lower baseline *Z*‐scores on all tests (difference: −0.097 to −0.592). Nevertheless, differences in longitudinal decline were non‐significant (annualized difference: −0.018 to 0.031).

**DISCUSSION:**

Despite baseline score differences, longitudinal change relative to clinical ratings appears comparable across racial groups.

**Highlights:**

- Version 3 of the NACC Uniform Data Set neuropsychological battery (UDS3) has been implemented since 2015 and administered to over 14,000 participants.
- We observed small differences in longitudinal decline across all test scores when comparing White and African American participants.
- The findings support the continued use of the UDS3 battery in ADRCs and its potential applicability in other studies of cognitive aging and decline.

## 1 BACKGROUND

The prevalence of Alzheimer's disease and related dementias (ADRD) is projected to increase over the next three decades^1^ given the rapidly growing older adult population. There is a relative dearth of information on risk of disease and progression of cognitive decline in ethnically diverse groups that are underrepresented in research.^2^ The National Alzheimer's Coordinating Center (NACC) at the University of Washington has coordinated data collection of the Uniform Data Set (UDS) on thousands of participants from over 35 past and current National Institute on Aging (NIA)‐funded Alzheimer's Disease Research Centers (ADRCs) since 2005.^3^ Participants across the spectrum of cognition, including no cognitive impairment, mild cognitive impairment (MCI), and dementia, are enrolled and followed annually. The UDS data include detailed clinical measurements and neuropsychological test scores for studying cognitive decline over time.

In 2015, the UDS version 3 (UDS3) neuropsychological battery was launched to replace some of the measures used in the earlier versions and to expand assessment to additional domains.^4^, ^5^ The instruments were constructed with the guidance and approval of the Clinical Task Force (CTF), a group formed by the NIA to develop standardized methods for collecting longitudinal data that would encourage and support collaboration across the ADRCs. The first published norms for the UDS3 neuropsychological battery did not present stratified analyses across race due to the relatively small sample size for African Americans collected between March 2015 and November 2016.^4^ Updated norms for African Americans have become available more recently using data available up to May 2019.^6^

Previous studies of racial differences in longitudinal cognitive test performance have yield mixed results. For example, in studies of African Americans and White Americans with a dementia diagnosis, racial differences have been found for both baseline and longitudinal decline.^7^, ^8^ In contrast, in studies of African Americans and White Americans without dementia, significant racial differences have been demonstrated in baseline levels of cognition,^9^, ^10^ with African Americans scoring lower, but results have been mixed for racial differences in decline.^11^, ^12^, ^13^, ^14^ The discrepant results for longitudinal studies could be due to the use of different neuropsychological batteries across studies. Given the widespread use of the UDS3, not only within the ADRCs but in other cohorts nationally^4^, ^5^ and internationally,^6^ understanding whether there are racial differences in decline on the UDS neuropsychological tests, especially as a function of diagnosis, is of interest for understanding the trajectory of aging and ADRD in different populations and for planning treatment trials that recruit diverse participants without cognitive impairment or among persons with MCI. The main purpose of the current study was to determine whether the UDS3 cognitive battery is comparably sensitive to longitudinal change between African American and White participants.

**RESEARCH IN CONTEXT**

1. **Systematic review**: The authors reviewed the literature using search engines such as PubMed. Previous studies used other batteries to demonstrate that there are significant racial differences between African Americans and White Americans in baseline levels of cognition, with mixed results for racial differences in decline. Racial difference in the longitudinal decline in neuropsychological test scores in the NACC's UDS3 is underexplored.
2. **Interpretation**: Our results suggest that longitudinal change appears comparable between White and African American participants, despite significant differences in baseline scores.
3. **Future directions**: Neuropsychological tests in the NACC's UDS3 are retained in version 4 to support the continued mission to understand factors affecting cognitive decline. The tests can be used in other studies and trials that include both White and African American participants. Studying the difference in other racial groups and test languages will be important future directions.

## 2 METHODS

### 2.1 Sample

The NACC data include study participants enrolled in the NIA‐funded ADRCs located across the United States. Each local ADRC obtained written informed consent from participants. Research using the NACC data was approved by the University of Washington Institutional Review Board. Participants in the individual centers reflect a mix of recruitment sources, including clinician referrals, self‐referrals by patients or family members, and community‐based recruitment to attract volunteers who wish to contribute to research. In addition, most centers also enroll persons with normal cognition. Therefore, NACC participants are not a representative sample of the U.S. population with or without dementia. Rather, they are best regarded as a referral‐based or volunteer case series selected based on each center's research focus. In addition, the ADRC participants are disproportionately of White racial background and highly educated,^1^ although recent efforts are being made to diversify the database. The current analytical sample was extracted from the September 2024 data freeze, which includes participants with baseline and follow‐up visits who were administered the English version of UDS3 neuropsychological tests during in‐person visits. We focus our analysis on participants who self‐reported White or African American racial background (including mixed race participants with primary self‐reported race as either White or African American), since the sample size for other race categories (American Indians, native Hawaiian, Asian, and others) were small or there was limited follow‐up (e.g., Hispanics tested in Spanish). We restricted analyses to participants with a baseline CDR global score less than 1 to take advantage of the long follow‐up and target the population most likely to benefit from prevention trials. We did not exclude participants based on physical or mental comorbidities. The analysis dataset contained participants with complete baseline observations on the variables listed in Table 1, which was 98.7% of the total White participants and 98.8% of African American participants with a baseline CDR global score less than 1.

**TABLE 1:** Descriptive characteristics of baseline sample by race.

	White Americans *N* = 11,534	African Americans *N* = 2855
Age		
<60	1694 (14.7%)	313 (11.0%)
60 to 69	3831 (33.2%)	1216 (42.6%)
70 to 79	4578 (39.7%)	1042 (36.5%)
≥80	1431 (12.4%)	284 (9.9%)
Sex		
Male	5202 (45.1%)	774 (27.1%)
Female	6332 (54.9%)	2081 (72.9%)
Education		
≤12	1259 (10.9%)	673 (23.6%)
13 to 15	1847 (16.0%)	739 (25.9%)
16	3222 (27.9%)	612 (21.4%)
≥17	5206 (45.2%)	831 (29.1%)
Global CDR		
0	6204 (53.8%)	1733 (60.7%)
0.5	5330 (46.2%)	1122 (39.3%)
Family history		
Yes	3687 (32.0%)	1073 (37.6%)
No	6303 (54.6%)	1174 (41.1%)
Unknown	1544(13.4%)	608 (21.3%)
Referral source		
Self/relative/friend	4500 (39.0%)	1391 (48.7%)
Health professional	6062 (52.6%)	1010 (35.4%)
Other	972 (8.4%)	454 (15.9%)
Diabetes		
Yes	1167 (10.1%)	762 (26.7%)
No	10367 (89.9%)	2093 (73.3%)
Hypertension		
Yes	4518 (39.2%)	1933 (67.7%)
No	7016 (60.8%)	922 (32.3%)
Cardiac events		
Yes	973 (8.4%)	199 (7.0%)
No	10561 (91.6%)	2656 (93.0%)

### 2.2 Outcome measures

The UDS3 neuropsychological test instruments are discussed in detail elsewhere.^4^ The outcome variables included the Montreal Cognitive Assessment (MoCA) for measuring overall cognitive impairment, Number Span (Forward/Backward) for measuring immediate memory/attention, Craft Story 21 Recall (Immediate/Delayed) for measuring episodic memory, Multilingual Naming Test (MINT) and Category Naming (animals and vegetables) for language, Trail Making A and B (total correct per minute) for psychomotor speed and executive function, respectively, and Benson Figure (Copy/Recall) for visuospatial function. *Z*‐scores for each test were constructed using published norms,^4^ which was based on baseline mean and standard deviations estimated using the normal controls (CDR score 0) in a combined White and African American sample. Additional analyses using published race‐specific norms^6^ for constructing the *Z*‐scores are given in the Supplementary Materials.

### 2.3 Statistical analysis

We described the baseline sample characteristics stratified by race. We estimated mean and standard deviation of baseline scores and tested the equality of observed scores across race by two‐sample *t* tests. Linear mixed‐effects models with subject‐specific random intercept were used to examine both the baseline differences and longitudinal changes in neuropsychological test scores. Among participants who are cognitively normal or have MCI at baseline, we investigated whether there were racial differences in neuropsychological scores among those who remain in their diagnostic categories based on CDR global scores or progressed into a higher CDR score within 5 years. We chose a 5‐year duration because it is a common study duration for trials and less than 10% of MCI participants in the NACC data have a follow‐up longer than 5 years. Further, the 5‐year cut‐off was applied only to exclude follow‐up measurements beyond 5 years for participants with longer follow‐up. Data from participants with less than 5 years of follow‐up were included in full, regardless of health conditions. Four diagnostic groups were considered (initial normal cognition without progression, initial normal cognition with progression, initial MCI without progression, and initial MCI with progression), and corresponding indicator variables were created. The diagnostic groups were created based on both baseline and all available longitudinal CDR measures for each participant. The progressor groups contained individuals who had consistent observed progression from the initial diagnosis, while participants with fluctuating or non‐progressive trajectories were categorized as non‐progressors. We performed a sensitivity analysis excluding participants from the progressor groups who had a higher CDR rating only at the last follow‐up visit, and the results did not show any notable deviation. All models included a racial group indicator (White vs. African American), time (in years), an interaction between time and racial group indicator, a baseline visit indicator, and an interaction between baseline visit and racial group indicators for modeling differential practice effects,^15^ age at initial visit, sex, years of education, diagnostic group indicators, and their interactions with time. This model specification allowed different baseline mean values and slopes across diagnostic groups. The interaction between time and racial groups quantified racial differences in change of scores over time. Model 1 included the independent variables given above. Two additional models progressively adjusted for additional baseline variables. Model 2 included all independent variables in Model 1 and added baseline comorbidities (diabetes, hypertension, cardiac events), and Model 3 further included recruitment‐based factors (family history and referral source). The indicator of cardiac events corresponded to the presence of at least one of the three conditions: myocardial infarction, atrial fibrillation, and congestive heart failure. A nominal significance level of 5% was used in describing the results. Bonferroni correction for multiple testing was utilized to offer a conservative family‐wise Type I error control over 28 simultaneous tests. Statistical analyses were performed using Stata version 14.2.

## 3 RESULTS

There were 14,389 participants recruited in 37 ADRCs who completed at least the baseline visit with complete information on age, education, sex, family history, referral source, and comorbidity conditions (diabetes, hypertension, cardiac events) with a baseline CDR global score less than one, of whom 2855 (19.8%) were African American. Table 1 shows the baseline sample characteristics stratified by race. Compared with White participants, African American participants were more likely to be female (73% vs 55%), had fewer years of education (proportion less than 16 years: 49% vs 27%), were more often cognitively normal at baseline (61% vs 54%), were less often referred by health professionals (35% vs 53%), and had a higher prevalence of diabetes (27% vs 10%) and hypertension (68% vs 39%). Table S1 shows the frequencies of baseline psychiatric morbidities.

Table 2 shows the summary statistics of the baseline neuropsychological test scores by race. Most test scores, except Vegetable List Generation and Benson Complex Figure (immediate recall), were significantly higher among White participants than African American participants.

**TABLE 2:** Summary statistics of Uniform Data Set version 3 neuropsychological test scores at baseline by race.

		Whites	African Americans	
Neuropsychological test scores	Domain	Mean (SD)	Mean (SD)	Difference in mean (95% CI)
Montreal Cognitive Assessment, total score	Dementia severity	24.40 (4.32)	23.21 (3.94)	1.19*** (1.02, 1.37)
Number Span Test Forward, total correct trials	Attention	7.93 (2.40)	7.44 (2.28)	0.49***(0.39, 0.59)
Number Span Test Forward, longest span	Attention	6.48 (1.36)	6.22 (1.30)	0.26*** (0.21, 0.32)
Number Span Test Backward, total correct trials	Attention	6.56 (2.29)	5.65 (2.12)	0.91*** (0.82, 1.01)
Number Span Test Backward, longest span	Attention	4.76 (1.33)	4.24 (1.23)	0.52*** (0.47, 0.57)
Craft Story 21 Recall Immediate Paraphrase, total units	Memory	14.00 (5.10)	13.50 (4.40)	0.50*** (0.29, 0.70)
Craft Story 21 Recall Delay Paraphrase, total units	Memory	12.33 (5.93)	11.69 (4.98)	0.64*** (0.40, 0.88)
Multilingual Naming Test (MINT), total score	Language naming	29.22 (3.74)	27.72 (2.91)	1.50*** (1.35, 1.65)
Animals List Generation, total in 60 s	Language category fluency	19.51 (6.38)	17.63 (5.09)	1.89*** (1.63, 2.14)
Vegetables List Generation, total in 60 s	Language category fluency	12.84 (4.88)	12.94 (4.08)	−0.09 (−0.29, 0.10)
Trail Making Test part A, total correct per minute	Psychomotor speed	47.94 (18.98)	40.11 (15.89)	7.83*** (7.07, 8.59)
Trail Making Test part B, total correct per minute	Executive function	18.77 (9.40)	14.56 (7.34)	4.21*** (3.83,4.60)
Benson Complex Figure Copy, total score	Visuospatial	15.15 (2.02)	14.84 (1.82)	0.31*** (0.22, 0.39)
Benson Complex Figure Recall, total score	Visuospatial/Memory	9.43 (4.25)	9.48 (3.77)	−0.06 (−0.23, 0.12)

*Note*: *P* value based on two‐sample *t* tests: *p < 0.05, **p < 0.01, ***p < 0.001.

Abbreviations: CI, confidence interval; SD, standard deviation.

Using primary models for each neuropsychological test, which incorporated both baseline and follow‐up data while adjusting for age, sex, education, comorbidities, and recruitment factors and allowing for differential cognitive decline across CDR categories, we estimated racial differences in test performance. Table 3 shows estimated baseline racial differences in *Z*‐scores, while Table 4 displays estimated differences in annualized change in *Z*‐scores by race. The three models presented in each table progressively adjust for relevant covariates. Generally speaking, the results are consistent across the three models, and in what follows we focus on Model 3, which adjusted for all specified covariates. Figure S1 shows the predicted mean trajectories from Model 3. At baseline, African American participants had lower scores than White participants across all tests, with estimated *Z*‐score differences ranging from −0.097 to −0.592. All the observed baseline differences were statistically significant, even after Bonferroni adjustment for multiple testing. In contrast, the differences in annualized cognitive decline between African American and White participants were small, with estimated differences ranging from −0.018 to 0.031. None of the 14 measures showed statistically significant racial differences in longitudinal decline with or without adjustment for multiple comparisons.

**TABLE 3:** Model‐based estimates of difference in baseline *Z*‐scores comparing African Americans with White Americans (reference group).

	African American vs White Estimate (95% C.I.)		
Neuropsychological test score	Model 1	Model 2	Model 3
Montreal Cognitive Assessment, total score	−0.483*** (−0.539, −0.426)	−0.482*** (−0.541, −0.424)	−0.487*** (−0.546, −0.427)
Number Span Test Forward, total correct trials	−0.163*** (−0.207, −0.119)	−0.134*** (−0.180, −0.089)	−0.134*** (−0.180, −0.089)
Number Span Test Forward, longest span	−0.158*** (−0.202, −0.114)	−0.130*** (−0.176, −0.085)	−0.133*** (−0.179, −0.087)
Number Span Test Backward, total correct trials	−0.387*** (−0.429, −0.345)	−0.360*** (−0.405, −0.316)	−0.360*** (−0.404, −0.316)
Number Span Test Backward, longest span	−0.372*** (−0.414, −0.331)	−0.348*** (−0.391, −0.305)	−0.348*** (−0.391, −0.304)
Craft Story 21 Recall Immediate Paraphrase, total units	−0.186*** (−0.231, −0.141)	−0.203*** (−0.249, −0.156)	−0.207*** (−0.254, −0.160)
Craft Story 21 Recall Delay Paraphrase, total units	−0.244*** (−0.292, −0.196)	−0.263*** (−0.313, −0.213)	−0.277*** (−0.327, −0.227)
Multilingual Naming Test (MINT), total score	−0.572*** (−0.639, −0.504)	−0.587*** (−0.657, −0.517)	−0.592*** (−0.663, −0.522)
Animals List Generation, total in 60 s	−0.344*** (−0.385, −0.303)	−0.330*** (−0.373, −0.288)	−0.343*** (−0.386, −0.300)
Vegetables List Generation, total in 60 s	−0.115*** (−0.156, −0.074)	−0.116*** (−0.158, −0.073)	−0.123*** (−0.166, −0.081)
Trail Making Test Part A, total correct per minute	−0.455*** (−0.494, −0.416)	−0.436*** (−0.476, −0.396)	−0.442*** (−0.483, −0.402)
Trail Making Test Part B, total correct per minute	−0.475*** (−0.514, −0.436)	−0.448*** (−0.489, −0.408)	−0.456*** (−0.497, −0.416)
Benson Complex Figure Copy, total score	−0.221*** (−0.285, −0.156)	−0.210*** (−0.277, −0.143)	−0.214*** (−0.281, −0.143)
Benson Complex Figure Recall, total score	−0.062* (−0.112, −0.013)	−0.081** (−0.133, −0.030)	−0.097*** (−0.149, −0.046)

*Note*: Model 1 adjusted for age, sex, education, allowing race‐dependent practice effects and different baseline mean and slope across Clinical Dementia Rating diagnostic groups. Model 2 included all independent variables in Model 1 and further adjusted for baseline comorbidities (diabetes, hypertension, cardiac events). Model 3 included all independent variables in Model 2 and further adjusted for family history and referral source. *P* value based on Wald tests: *p < 0.05, **p < 0.01, ***p < 0.001.

Abbreviation: CI, confidence interval.

**TABLE 4:** Model‐based estimates of the difference in longitudinal change in *Z*‐scores comparing African Americans with White Americans (reference group).

	Difference in annualized change estimate (95% CI)		
Neuropsychological test score	Model 1	Model 2	Model 3
Montreal Cognitive Assessment, total score	0.031 (−0.003, 0.064)	0.031 (−0.003, 0.064)	0.031 (−0.003, 0.065)
Number Span Test Forward, total correct trials	−0.012 (−0.039, 0.014)	−0.012 (−0.038, 0.014)	−0.012 (−0.038, 0.014)
Number Span Test Forward, longest span	−0.018 (−0.048, 0.011)	−0.018 (−0.048, 0.012)	−0.018 (−0.048, 0.012)
Number Span Test Backward, total correct trials	0.006 (−0.021, 0.033)	0.006 (−0.021, 0.034)	0.006 (−0.021, 0.034)
Number Span Test Backward, longest span	0.018 (−0.012, 0.047)	0.018 (−0.012, 0.048)	0.018 (−0.012, 0.048)
Craft Story 21 Recall Immediate Paraphrase, total units	0.005 (−0.024, 0.034)	0.005 (−0.024, 0.034)	0.005 (−0.024, 0.034)
Craft Story 21 Recall Delay Paraphrase, total units	0.015 (−0.014, 0.044)	0.015 (−0.014, 0.044)	0.015 (−0.014, 0.044)
Multilingual Naming Test (MINT), total score	0.016 (−0.017, 0.049)	0.016 (−0.017, 0.049)	0.017 (−0.013, 0.050)
Animals List Generation, total in 60 s	0.024 (−0.002, 0.049)	0.024 (−0.002, 0.049)	0.024 (−0.001, 0.050)
Vegetables List Generation, total in 60 s	0.008 (−0.019, 0.036)	0.008 (−0.019, 0.036)	0.009 (−0.018, 0.036)
Trail Making Test Part A, total correct per minute	0.000 (−0.024, 0.025)	0.000 (−0.024, 0.025)	0.000 (−0.024, 0.025)
Trail Making Test Part B, total correct per minute	0.005 (−0.018, 0.029)	0.005 (−0.019, 0.029)	0.005 (−0.018, 0.029)
Benson Complex Figure Copy, total score	0.020 (−0.027, 0.066)	0.020 (−0.027, 0.066)	0.020 (−0.027, 0.067)
Benson Complex Figure Recall, total score	0.025 (−0.005, 0.055)	0.025 (−0.005, 0.055)	0.026 (−0.005, 0.056)

*Note*: Model 1 adjusted for age, sex, and education, allowing race‐dependent practice effects and different baseline mean and slope across CDR diagnostic groups. Model 2 included all independent variables in Model 1 and further adjusted for baseline comorbidities (diabetes, hypertension, cardiac events). Model 3 included all independent variables in Model 2 and further adjusted for family history and referral source. *P* value based on Wald tests: *p < 0.05, **p < 0.01, ***p < 0.001.

Abbreviation: CI, confidence interval.

Tables S2 and S3 present results based on race‐specific *Z*‐scores, constructed using published race‐specific means and standard deviations, with the same model specification as in Model 3 of Tables 3 and 4. These analyses assess whether the observed effects in Tables 3 and 4 are robust to different scaling of the outcomes. The results for racial differences in longitudinal decline remained largely unchanged, but we still observed significant baseline differences consistent with previous literature.

## 4 DISCUSSION

Using UDS3 neuropsychological data from over 35 ADRCs representing over 14,000 participants, we examined racial differences in baseline and longitudinal decline among White and African American participants with a baseline CDR of less than one. African American participants had lower baseline neuropsychological test scores compared to White participants, but differences in longitudinal decline were small and insignificant. Our results suggest the UDS3 battery has comparable sensitivity to longitudinal changes across race relative to clinically ascertained functional status.

In a large observational cohort such as NACC, data collection often requires balancing multiple objectives to allow a wide variety of scientific questions to be studied. In 2020, during the development of UDS version 4 (UDS4), the CTF raised concerns about whether UDS3 tests were equally applicable across racial groups. A preliminary analysis using data collected up to that time yielded conclusions similar to those presented here – that UDS3 tests are effective in measuring cognitive changes across racial groups.^16^ This contributed to the CTF's recommendation to retain the UDS3 battery in UDS4 (which includes the addition of a verbal learning test). Our current analysis, which draws on a sample approximately twice the size of the earlier analysis, continues to suggest that the utility of the UDS3 battery for assessing longitudinal change appears comparable across African American and White samples. In 2017, the Spanish‐language version of UDS3 was introduced and Hispanic enrollment has recently increased. However, the volume of follow‐up data for Spanish tests is not yet sufficient for accurate longitudinal comparisons and will be an important future research direction.

Studying performance of assessment instruments for longitudinal decline in cognitive function is important for clinical trials and for clinical care regarding the spectrum of cognitive aging from normal to dementia, but obtaining high‐quality longitudinal research data with large sample sizes that are ethnically diverse can be challenging for any single study. The NACC cohort offers a unique resource in this regard, since annual neuropsychological test scores were collected from more than 35 ADRCs following a common UDS protocol for many years. Our results also have important implications for future prevention trials that use the UDS3 (or UDS4) battery as the outcome to assess change in cognitive function over time, supporting the inclusion of both White and African Americans in trial designs, as the battery has comparable sensitivity to longitudinal change across racial groups.

Using a population‐based cohort and different cognitive tests, similar *Z*‐score differences in baseline measures between African American and White participants have been observed.^13^ While it has been suggested that such observed differences could be partially explained by the difference in education levels between White and African American participants,^13^ our research shows a sizable residual unexplained racial difference in the cognitive measures after adjusting for years of education and other covariates. To aid the interpretation of these *Z*‐score differences that reflect standardized mean differences of the test scores, we back‐translated the differences estimated in Model 3 to raw scores and provided a sample size requirement for a clinical trial to detect the observed differences across two arms in Table S4. In addition, we compared the baseline *Z*‐score differences across race with the differences between participants with normal cognition without progression and those with MCI without progression. The results are given in Table S5. In 10 of the 14 measures, the baseline racial differences were less than 50% of the magnitude of the baseline diagnostic group differences. Table S6 further compares the baseline racial differences with the Z‐score changes associated with three additional years of education. In 11 of the 14 measures, three additional years of education accounted for at least 50% of the observed racial differences.

Race represents an incomplete proxy for other sociocultural variables such as quality of education, socioeconomic status, experiences of discrimination, and neighborhood disadvantage,^2^ which are likely factors contributing to racial differences in observed scores. These factors are not currently measured in the NACC UDS3 dataset, which limits the interpretive depth for any racial comparisons, including the current study, although the primary purpose of the present study was not to explain the difference on test performance between African American and White participants. UDS4, launched recently in January 2025, includes a new Social Determinants of Health module^17^ capturing 38 additional measures on five domains such as access to transportation, financial and environmental factors, healthcare experiences, and discrimination. This module may help address these important gaps in future racial comparison studies.

Several other limitations of this study should be acknowledged. First, although the African American sample has grown substantially, other racial and ethnic groups, such as Hispanic and Asian American participants, remain underrepresented and were not included in the current analysis. Thus, our findings do not generalize to other racial or ethnic groups and may not apply to a specific subpopulation, such as African Americans with less than a high‐school education. Second, like many aging and Alzheimer's focused studies, the ADRCs are biased toward highly educated participants. While we adjusted for education, we were unable to account for quality of education, early‐life socioeconomic status, and other important factors that may help explain the difference in baseline test scores. Third, the reliance on volunteer‐based recruitment in ADRCs and center‐specific recruitment strategies may introduce selection bias, as participants may not be representative of the broader population. As demonstrated previously,^18^ participants racialized as White and African American in the NACC data set have differential selection, which may have influenced baseline and longitudinal results. Although UDS3 includes a variable for source of recruitment as clinic referral, the “non‐clinic referral” group aggregates several heterogeneous referral sources (e.g., self‐referral, family referral, community outreach, and other non‐professional contacts), making it difficult to understand the potential confounding effects due to recruitment source. It will be important for ADRCs to make greater efforts to recruit older adults that represent understudied segments of the population, including those with lower education, other racial/ethnic groups, and those whose primary language is not English.

A major strength of this study is the large and geographically diverse sample drawn from over 35 ADRCs, all using a standardized and well‐established neuropsychological test battery. The availability of harmonized data across sites, combined with growing racial diversity in the cohort, enabled meaningful longitudinal comparisons of the UDS3 neuropsychological battery between African American and White participants.

## CONFLICT OF INTEREST STATEMENT

The authors report no conflicts of interest with regard to this manuscript. The funders had no role in study conception, design, or writing of this manuscript. Author disclosures are available in the Supporting Information.

## CONSENT STATEMENT

Research using the NACC data was approved by the University of Washington Institutional Review Board (IRB). All contributing ADRCs are required to obtain informed consent from their participants and maintain their own local IRB reviews and approvals prior to submitting data to NACC.

## Supporting information



Supporting Information

Supporting Information

## References

[1] Rajan KB , Weuve J , Barnes LL , McAninch EA , Wilson RS , Evans DA . Population estimate of people with clinical Alzheimer's disease and mild cognitive impairment in the United States (2020‐2060). Alzheimers Dement. 2021;17(12):1966‐1975. doi:10.1002/alz.12362 34043283 PMC9013315

[2] Babulal GM , Quiroz YT , Albensi BC , et al. Perspectives on ethnic and racial disparities in Alzheimer's disease and related dementias: update and areas of immediate need. Alzheimers Dement. 2019;15(2):292‐312. doi:10.1016/j.jalz.2018.09.009 PMC6368893 30555031

[3] Morris JC , Weintraub S , Chui HC , et al. The Uniform Data Set (UDS): clinical and cognitive variables and descriptive data from Alzheimer Disease Centers. Alzheimer Dis Assoc Disord. 2006;20(4):210‐216. doi:10.1097/01.wad.0000213865.09806.92 17132964

[4] Weintraub S , Besser L , Dodge HH , et al. Version 3 of the Alzheimer Disease Centers' Neuropsychological Test Battery in the Uniform Data Set (UDS). Alzheimer Dis Assoc Disord. 2018;32(1):10‐17. doi:10.1097/WAD.0000000000000223 29240561 PMC5821520

[5] Dodge HH , Goldstein FC , Wakim NI , et al. Differentiating among stages of cognitive impairment in aging: version 3 of the Uniform Data Set (UDS) neuropsychological test battery and MoCA index scores. Alzheimers Dement (N Y). 2020;6(1):e12103. doi:10.1002/trc2.12103 33283037 PMC7683960

[6] Sachs BC , Steenland K , Zhao L , et al. Expanded Demographic Norms for Version 3 of the Alzheimer Disease Centers' Neuropsychological Test Battery in the Uniform Data Set. Alzheimer Dis Assoc Disord. 2020;34(3):191‐197. doi:10.1097/WAD.0000000000000388 PMC7842186 32483017

[7] Barnes LL , Wilson RS , Li Y , et al. Racial differences in the progression of cognitive decline in Alzheimer disease. Am J Geriatr Psychiatry. 2005;13(11):959‐967. doi:10.1176/appi.ajgp.13.11.959 16286439

[8] Barnes LL , Wilson RS , Li Y , Gilley DW , Bennett DA , Evans DA . Change in cognitive function in Alzheimer's disease in African‐American and white persons. Neuroepidemiology. 2006;26(1):16‐22. doi:10.1159/000089231 16254449

[9] Werry AE , Daniel M , Bergström B . Group differences in normal neuropsychological test performance for older non‐Hispanic White and Black/African American adults. Neuropsychology. 2019;33(8):1089‐1100. doi:10.1037/neu0000579 PMC6823108 31343234

[10] Mehta KM , Simonsick EM , Rooks R , et al. Black and white differences in cognitive function test scores: what explains the difference?. J Am Geriatr Soc. 2004;52(12):2120‐2127. doi:10.1111/j.1532-5415.2004.52575.x PMC2939725 15571554

[11] Castora‐Binkley M , Peronto CL , Edwards JD , Small BJ . A longitudinal analysis of the influence of race on cognitive performance. J Gerontol B Psychol Sci Soc Sci. 2015;70(4):512‐518. doi:10.1093/geronb/gbt112 24184780

[12] Byrd DR , Gee GC , Tarraf W . Black‐white mental status trajectories: what ages do differences emerge?. SSM Popul Health. 2018;6:169‐177. doi:10.1016/j.ssmph.2018.09.008 30310849 PMC6178241

[13] Weuve J , Barnes LL , Mendes de Leon CF , et al. Cognitive Aging in Black and White Americans: cognition, Cognitive Decline, and Incidence of Alzheimer Disease Dementia. Epidemiology. 2018;29(1):151‐159. doi:10.1097/EDE.0000000000000747 28863046 PMC5718953

[14] Yang YC , Walsh CE , Shartle K , et al. An Early and Unequal Decline: life Course Trajectories of Cognitive Aging in the United States. J Aging Health. 2024;36(3‐4):230‐245. doi:10.1177/08982643231184593 PMC10728348 37335551

[15] Vivot A , Power MC , Glymour MM , et al. Jump, Hop, or Skip: modeling Practice Effects in Studies of Determinants of Cognitive Change in Older Adults. Am J Epidemiol. 2016;183(4):302‐314. doi:10.1093/aje/kwv212 26825924 PMC4753282

[16] Chan KCG , Barnes LL , Saykin AJ , et al. Racial‐ethnic differences in baseline and longitudinal change in neuropsychological test scores in the NACC Uniform Data Set 3.0. Alzheimer's & Dementia. 2021;17:e054653.

[17] Zuelsdorff M , Abner EL , Balls‐Berry JE , et al. Introducing social determinants of health to the Alzheimer's Disease Research Center network: development and implementation in the Uniform Data Set. Alzheimers Dement. 2025;21(5):e70279. doi:10.1002/alz.70279 40407095 PMC12100502

[18] Gleason CE , Norton D , Zuelsdorff M , et al. Association between enrollment factors and incident cognitive impairment in Blacks and Whites: data from the Alzheimer's Disease Center. Alzheimers Dement. 2019;15(12):1533‐1545. doi:10.1016/j.jalz.2019.07.015 PMC6925619 31601516

