# Supplementary material for: Comparable performance of the NACC Uniform Data Set version 3 neuropsychological test battery in assessing longitudinal cognitive change for African American and White participants
Source: Alzheimers Dement. 2025 Nov 8;21(11):e70889. doi: 10.1002/alz.70889 (PMC12596166; doi:10.1002/alz.70889)
Supplement: Supplementary file 1 — Supporting Information [file ALZ-21-e70889-s002.docx]

Supplementary Table 1. Psychiatric morbidities of the Baseline Sample by Race.

|  |  | White  Americans  N=11534 | African Americans  N=2855 |
| --- | --- | --- | --- |
| Depression | Yes | 1517 (13.2%) | 257 (9.0%) |
|  | No | 10017 (86.8%) | 2598 (91.0%) |
|  |  |  |  |
| TBI | Yes | 2035 (17.9%) | 301 (10.5%) |
|  | No | 9404 (81.5%) | 2536 (88.8%) |
|  | Missing | 95 (0.9%) | 18 (0.7%) |
|  |  |  |  |
| Seizures | Yes | 245 (2.2%) | 84 (2.9%) |
|  | No | 11227 (97.3%) | 2764 (96.8%) |
|  | Missing | 62 (0.5%) | 7 (0.3%) |
|  |  |  |  |
| Cardiac events | Yes | 647 (5.6%) | 273 (9.6%) |
|  | No | 10887 (94.4%) | 2582 (90.4%) |

Supplementary Figure 1. Predicted mean trajectory from Model 3 over five years comparing White and African American participants who were male aged 65 at baseline, 16 years of education, without diabetes, hypertension and cardiac conditions at baseline, without family history and referred by non-professional contacts, had normal cognition (CDR=0) at baseline and progressed to CDR>0 within 5 years.

| 1. MOCA   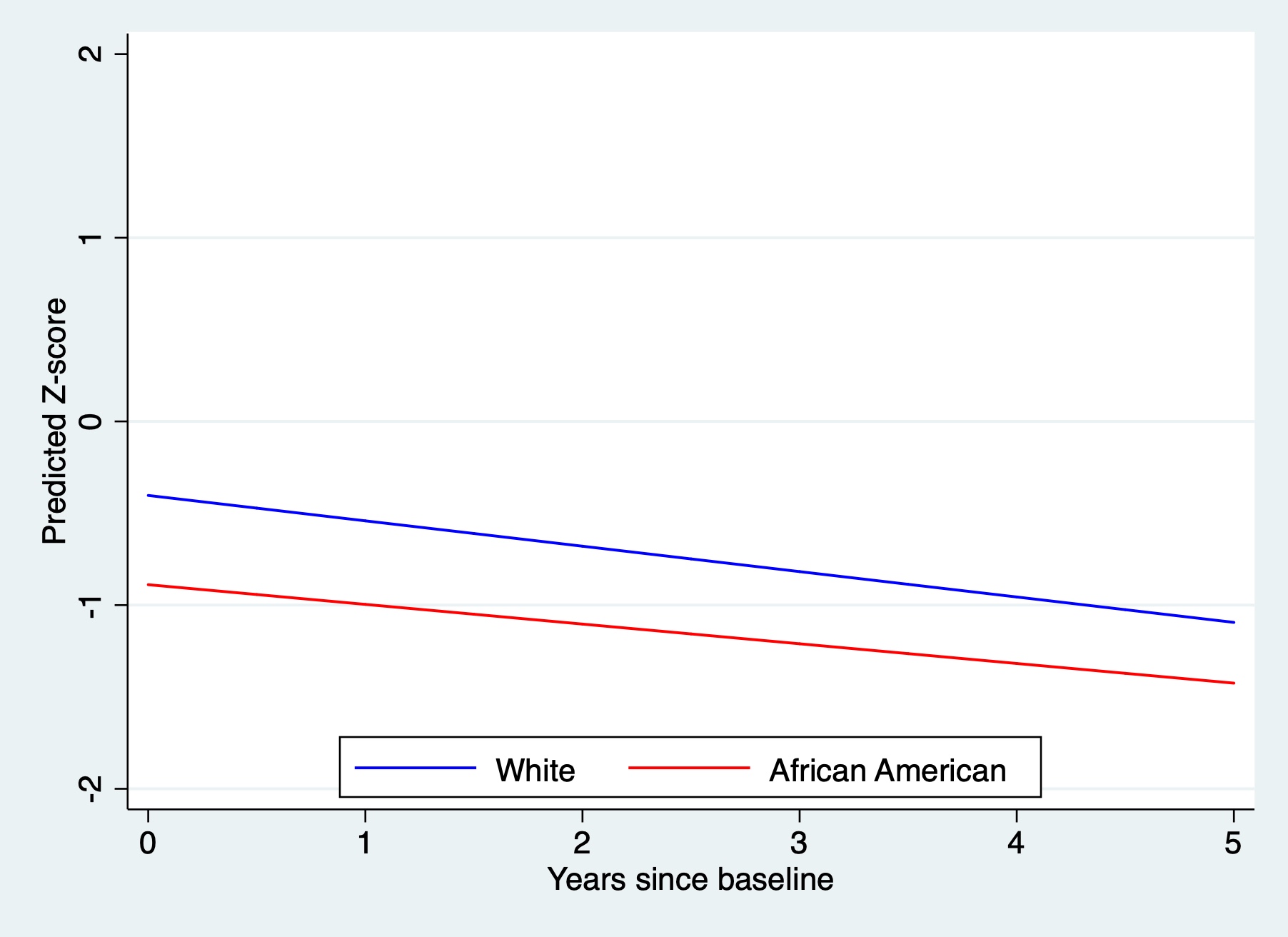 | 1. Number Span Test Forward, total correct trials   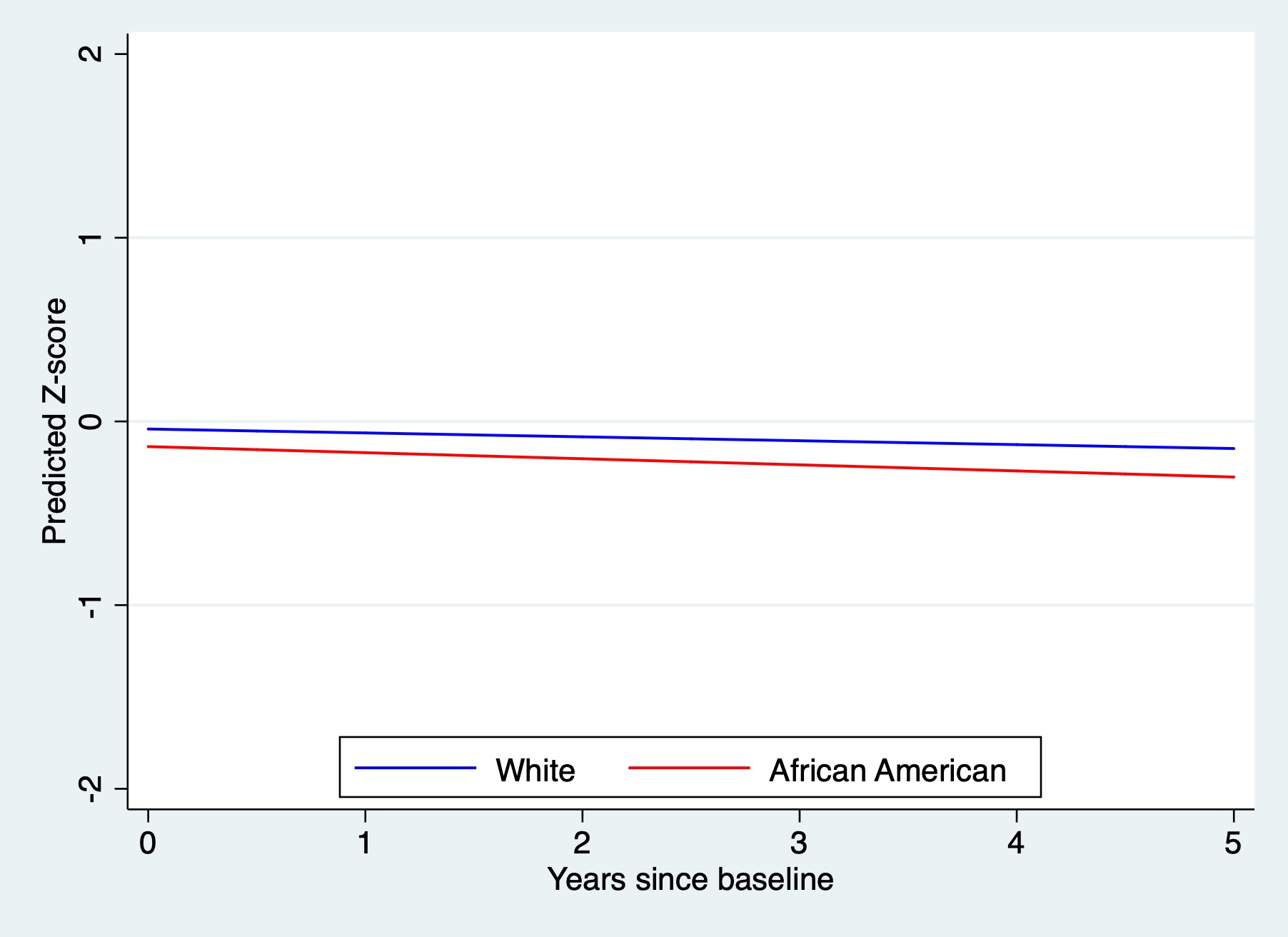 |
| --- | --- |
| 1. Number Span Test Forward, longest span   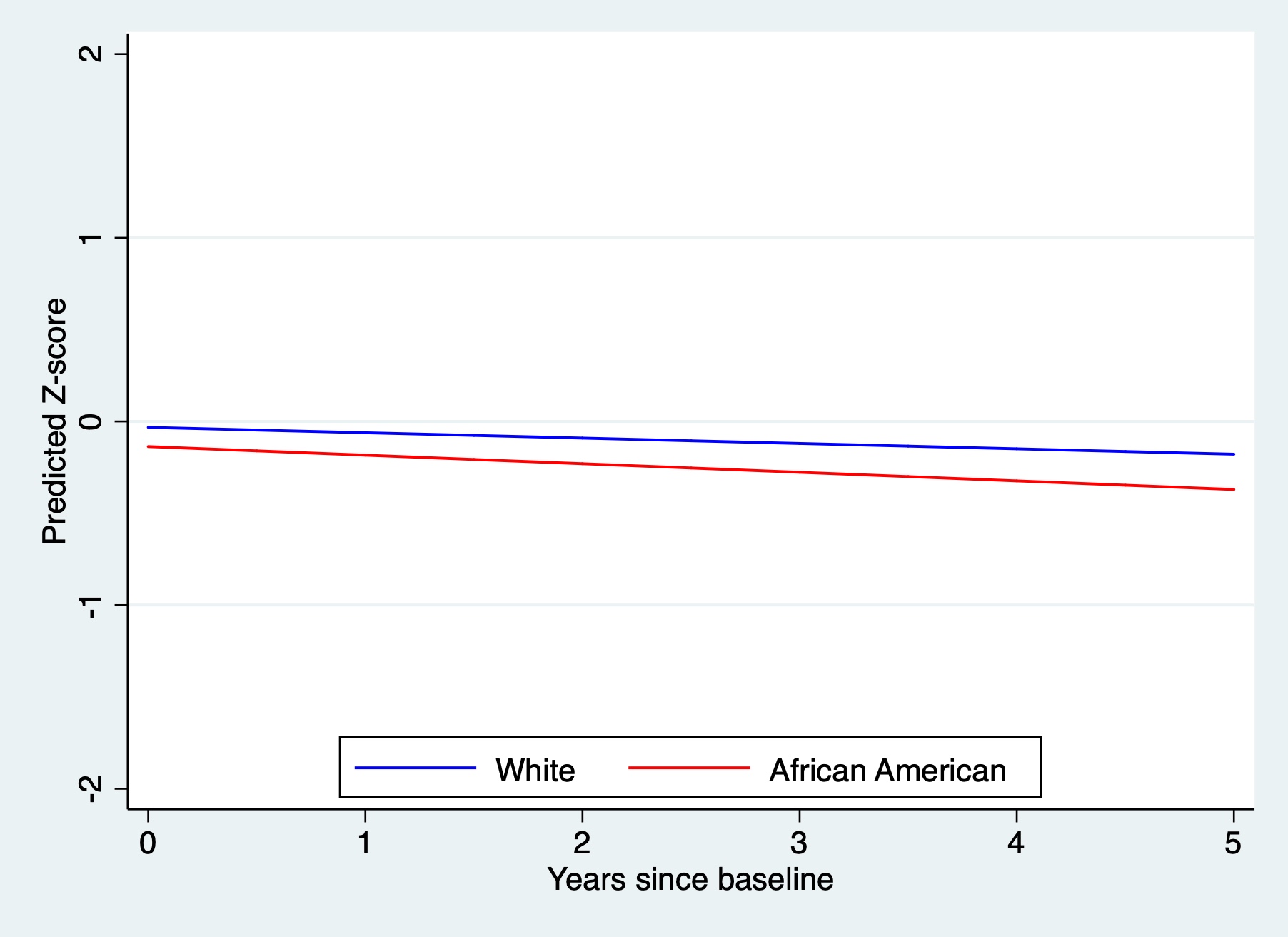 | 1. Number Span Test Backward, total correct trials   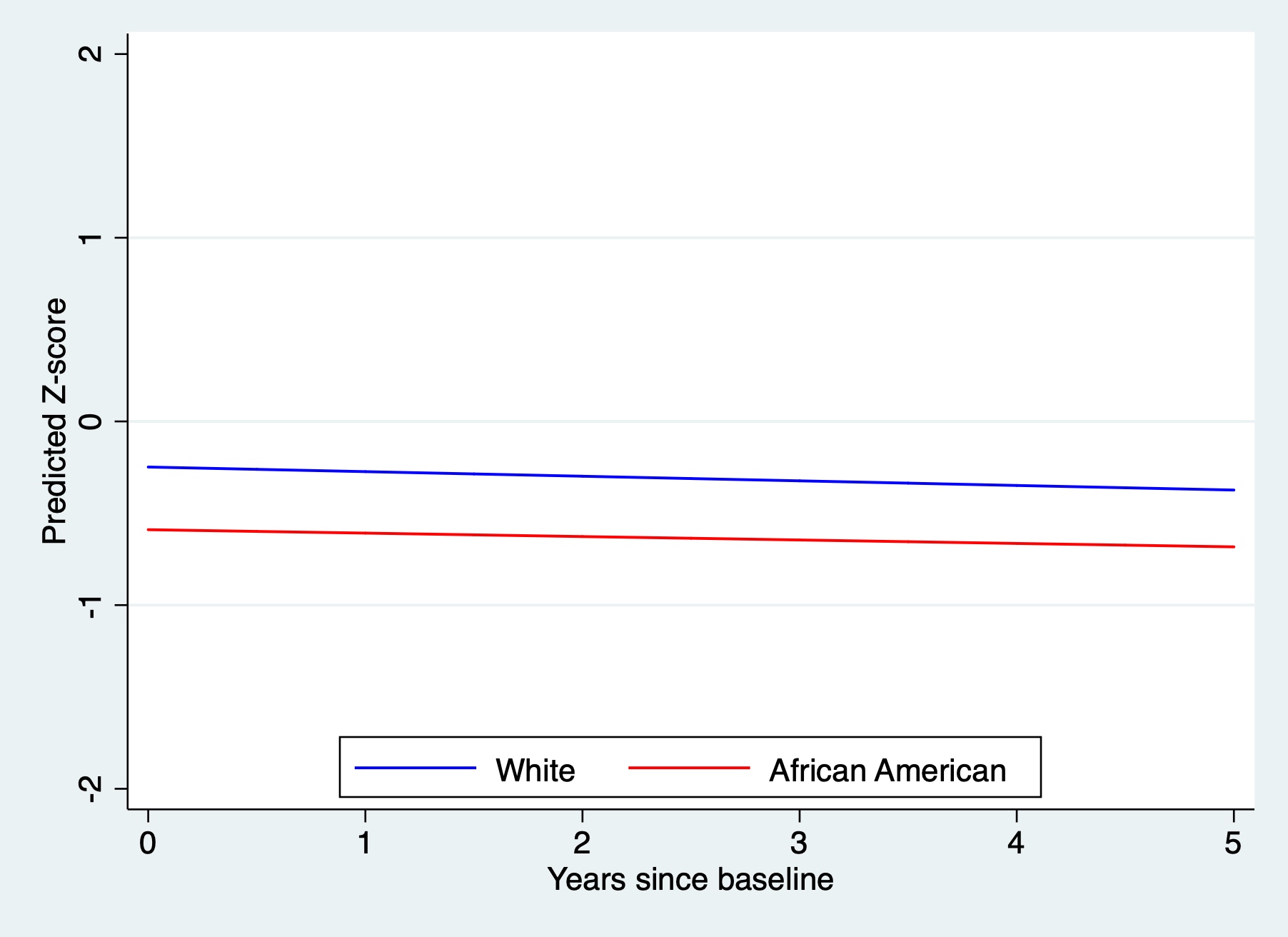 |
| 1. Number Span Test Backward, longest span   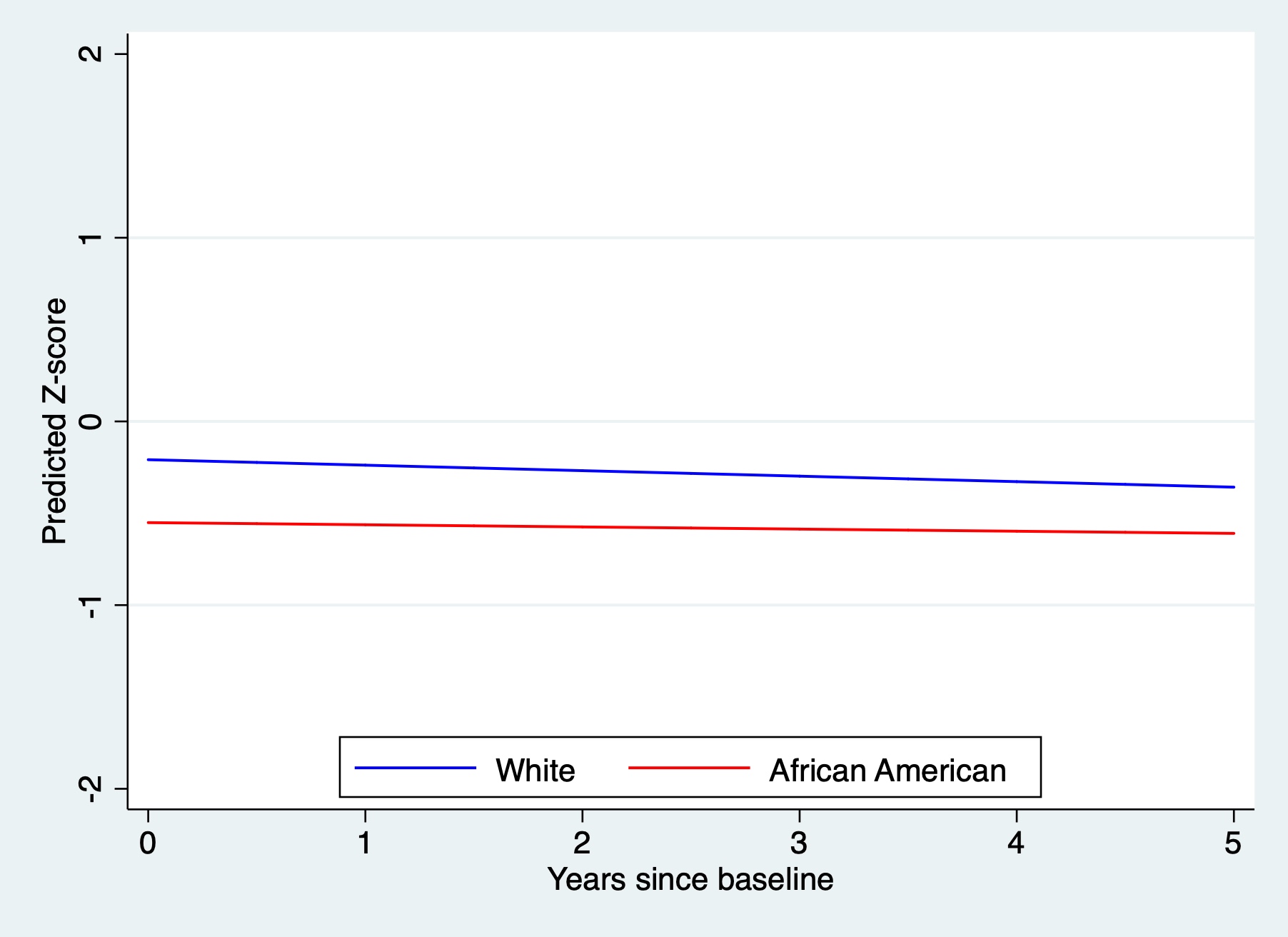 | 1. Craft Story 21 Recall Immediate Paraphrase, total units   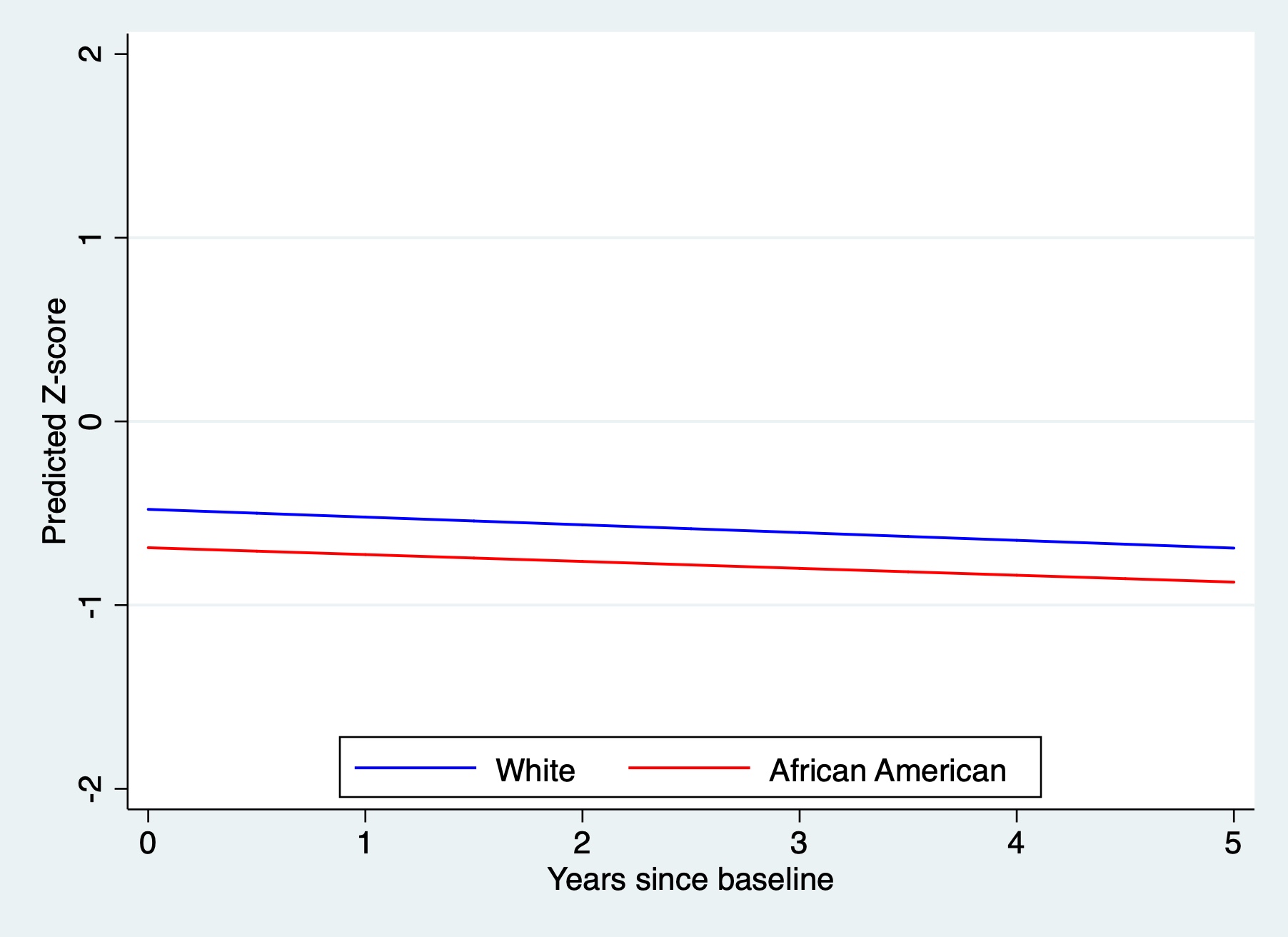 |
| 1. Craft Story 21 Recall Delay Paraphrase, total units   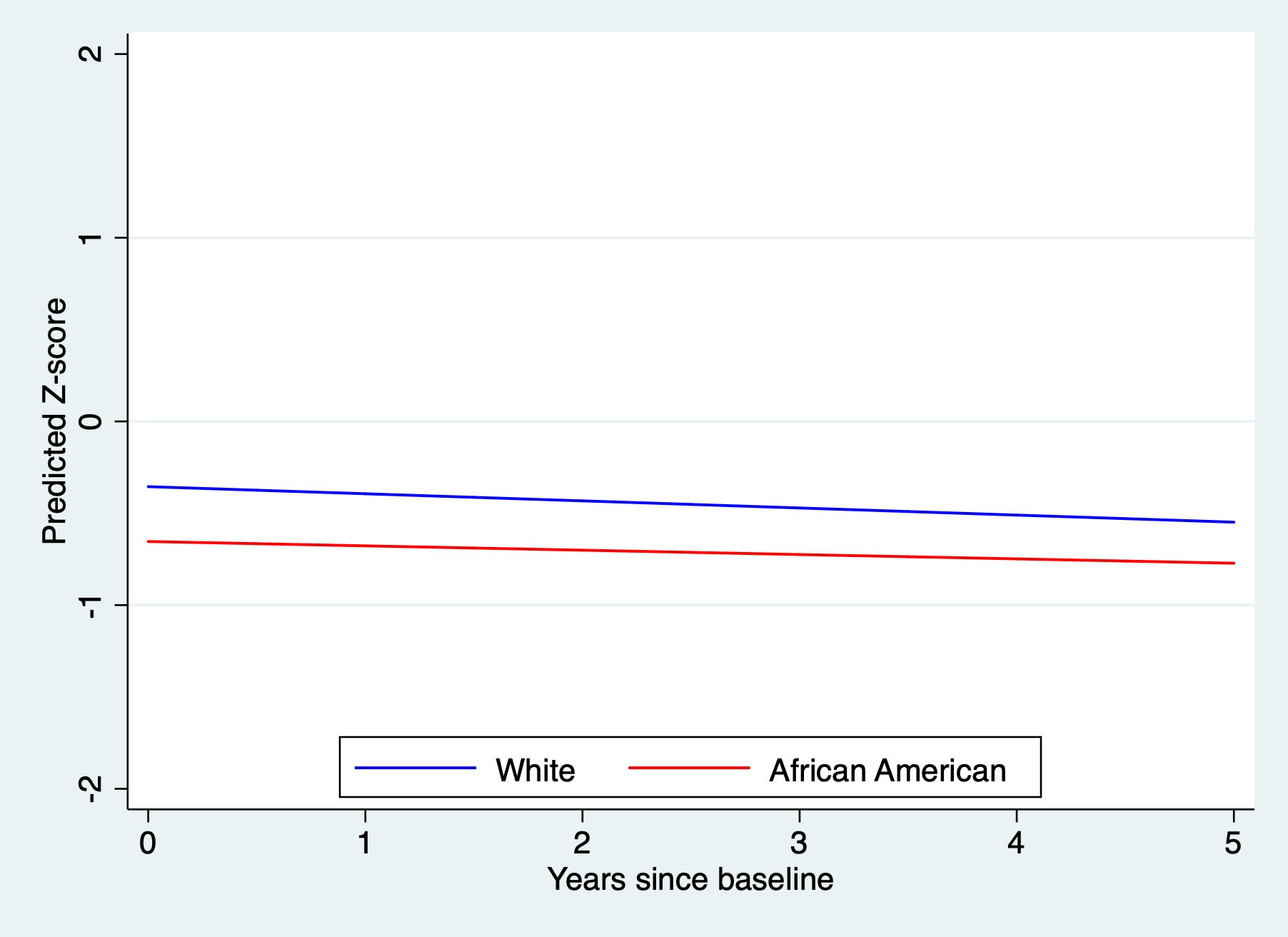 | 1. Multilingual Naming Test (MINT), total score   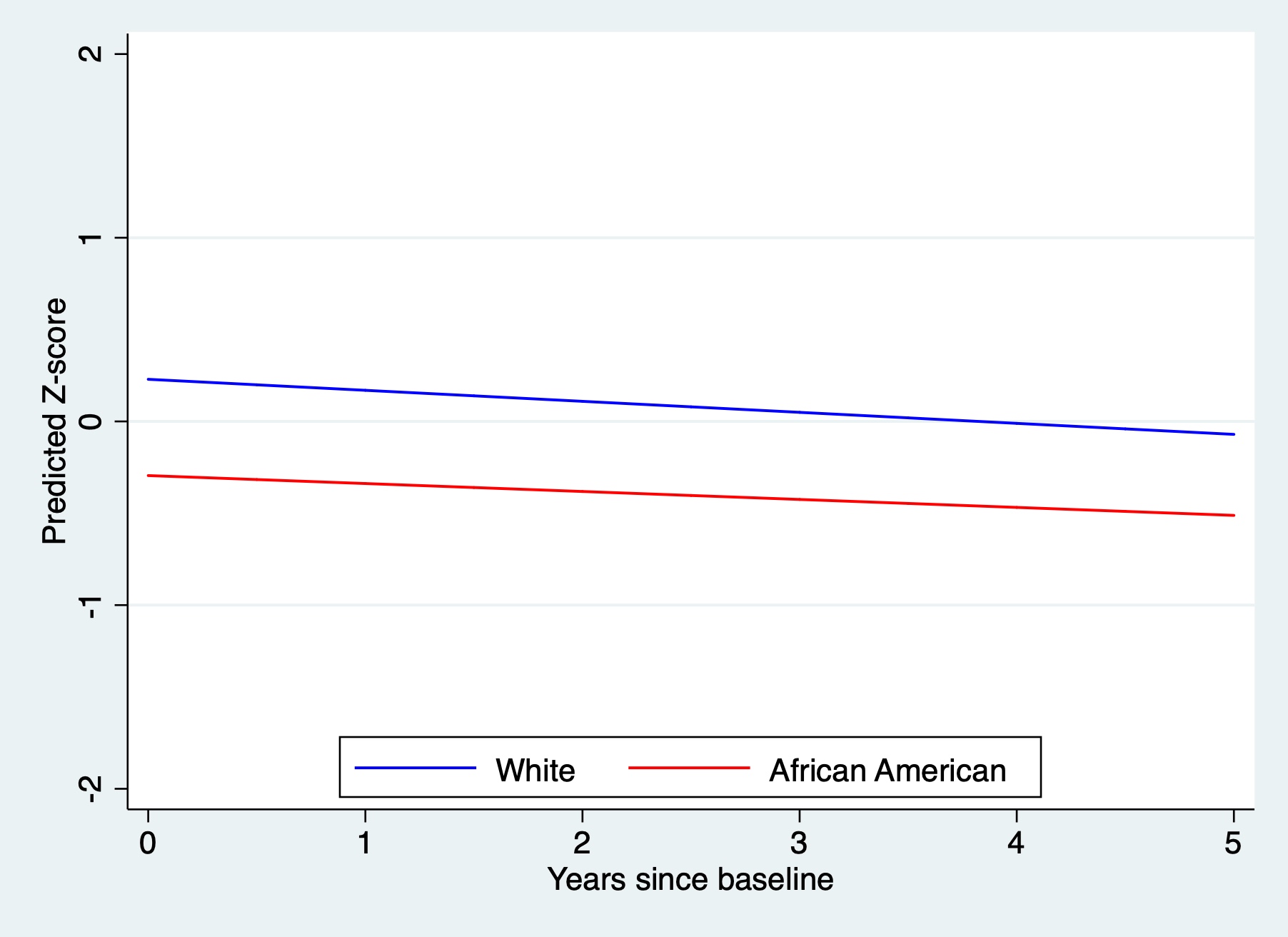 |
| 1. Animals List Generation, total in 60s   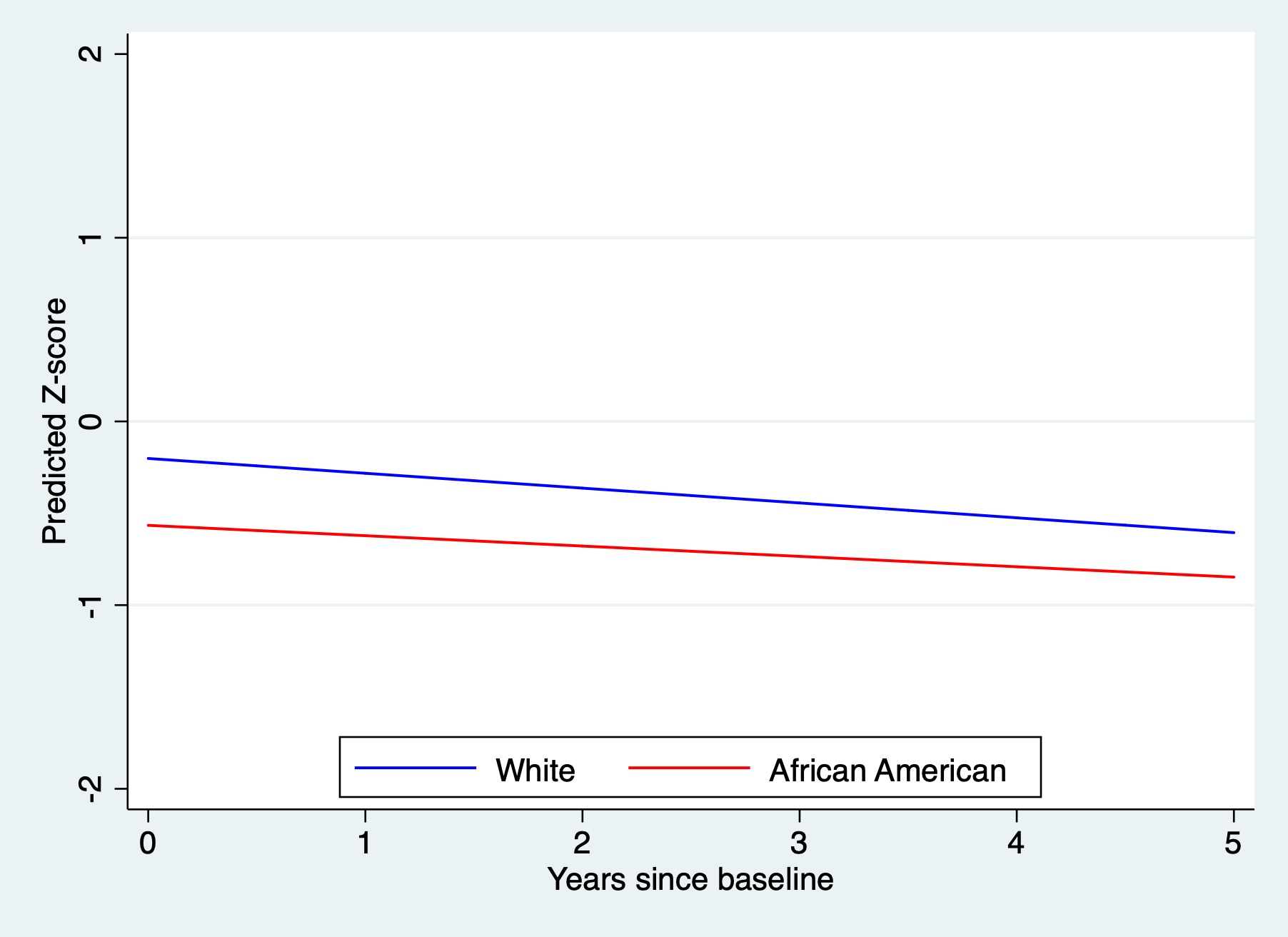 | 1. Vegetables List Generation, total in 60s   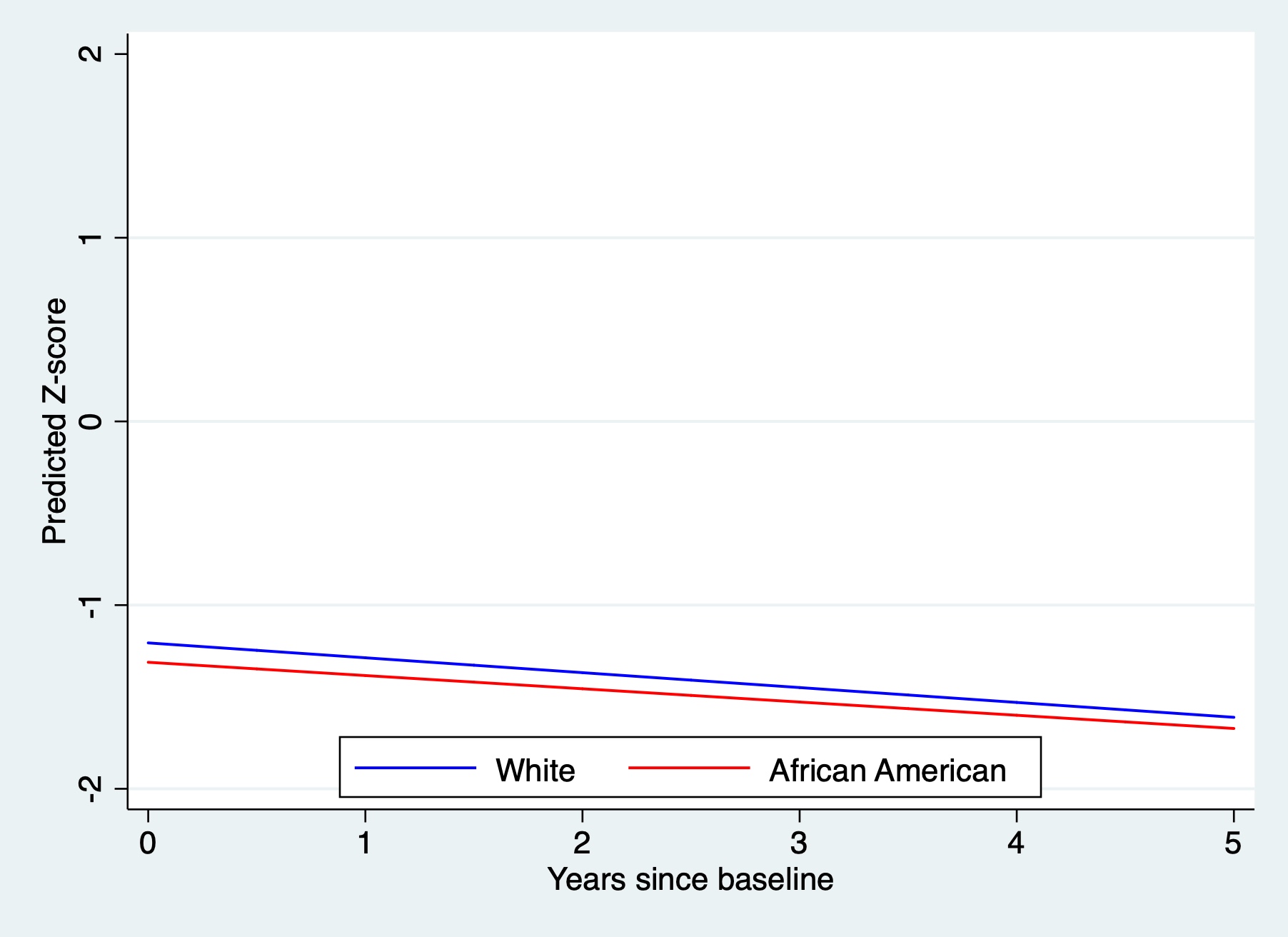 |
| 1. Trail Making Test part A, total correct per minute   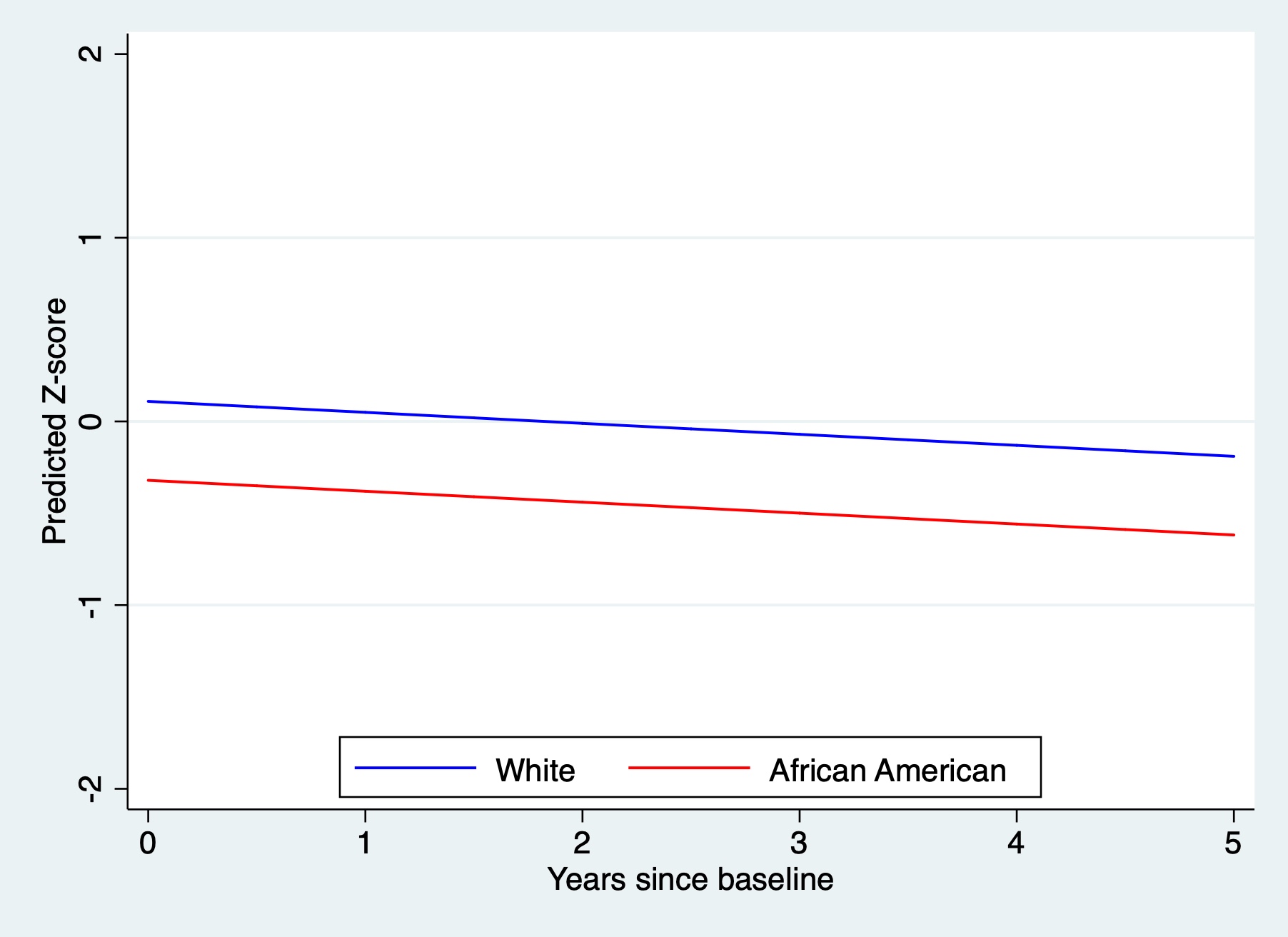 | 1. Trail Making Test part B, total correct per minute   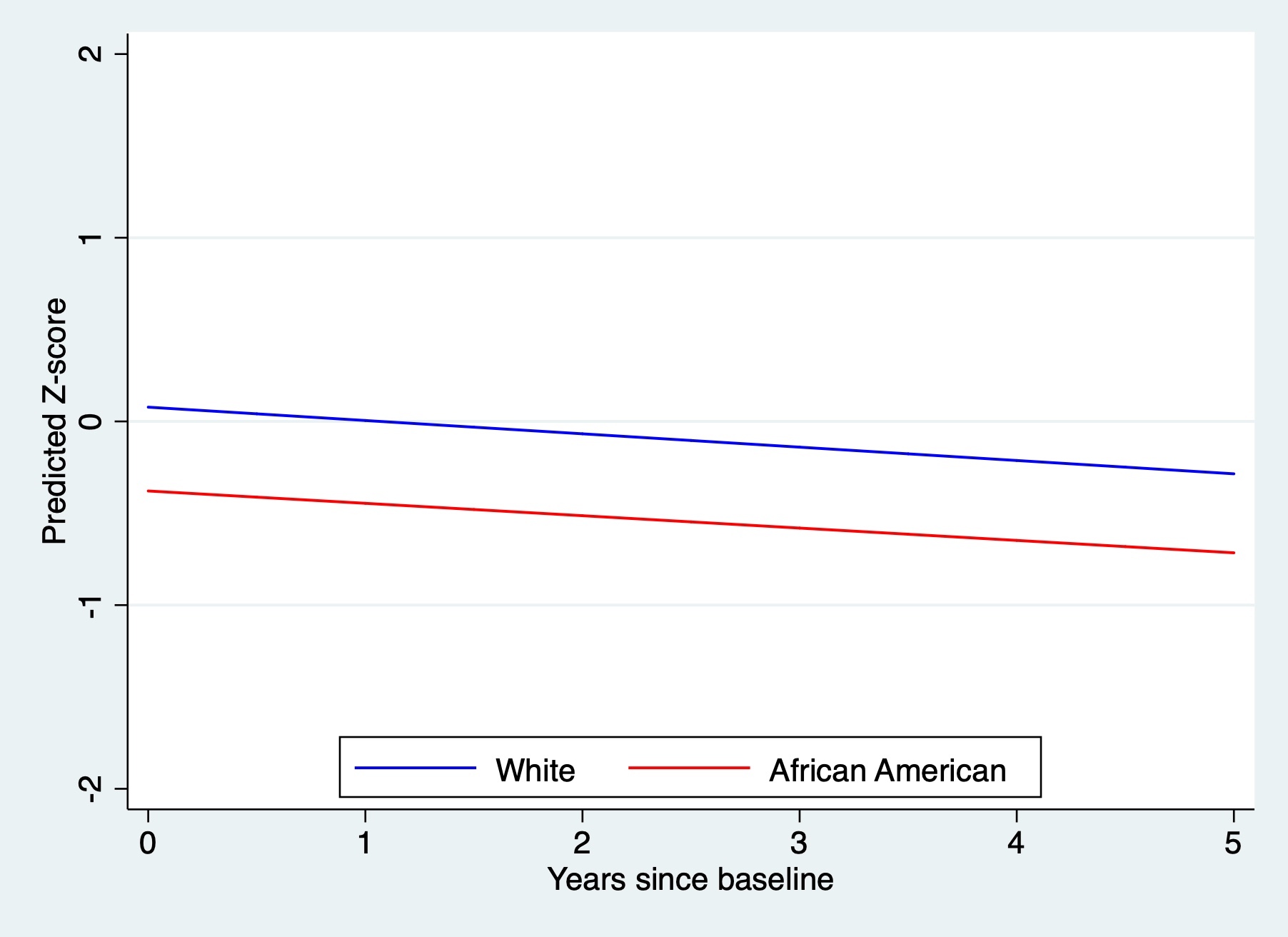 |
| 1. Benson Complex Figure Copy, total score   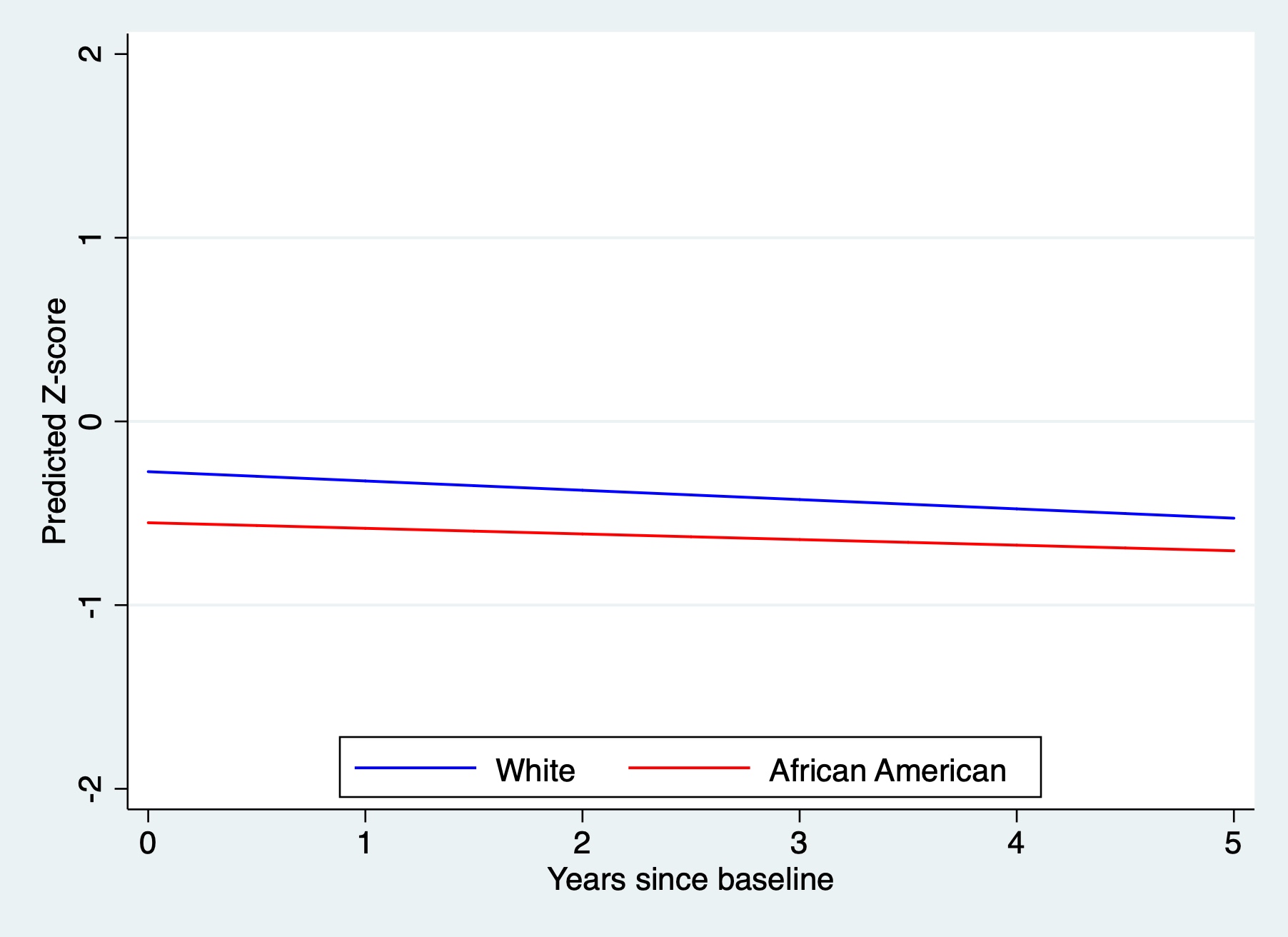 | 1. Benson Complex Figure Recall, total score   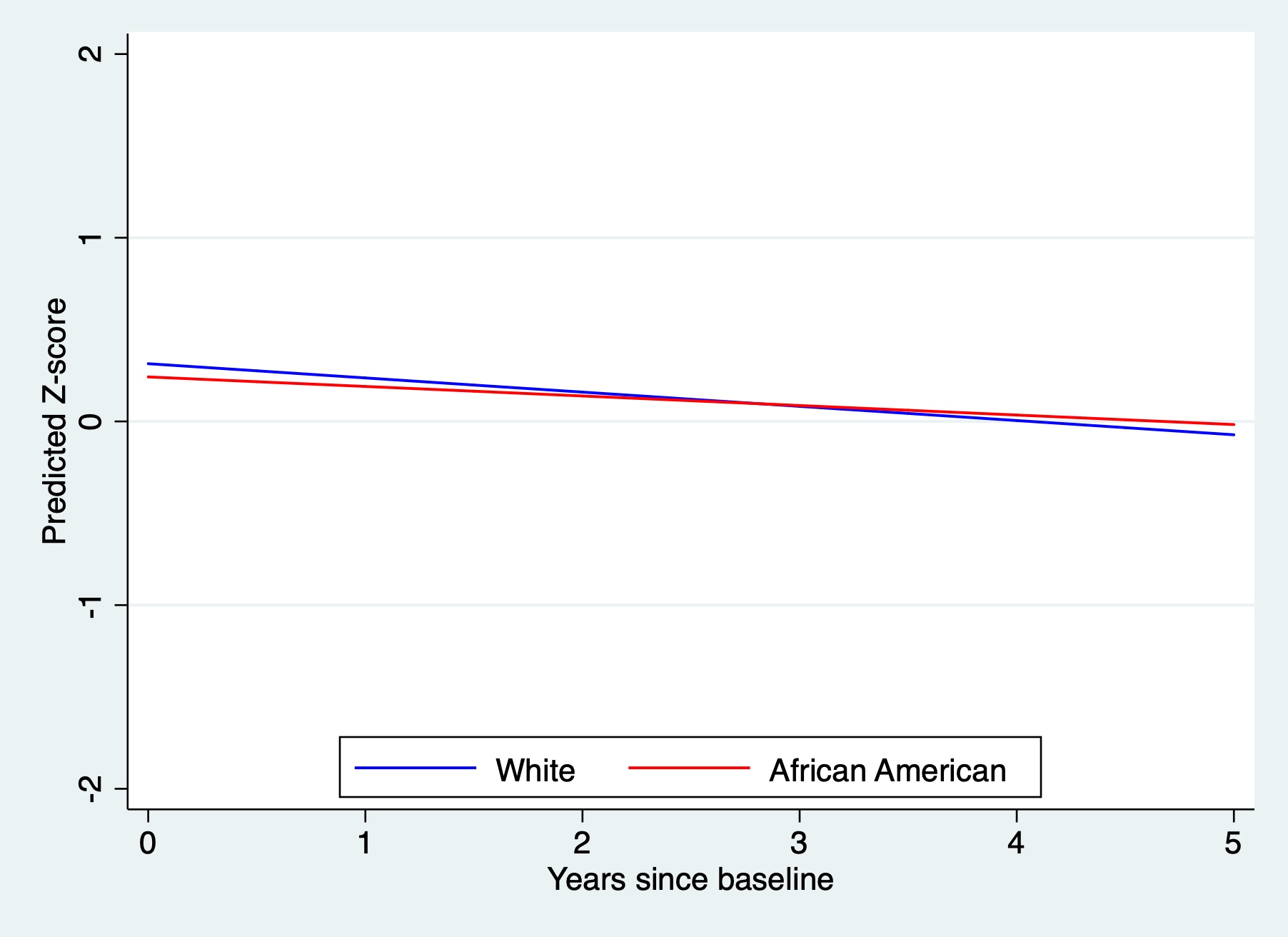 |

Supplementary Table 2. Model-based Estimates of the Difference in Baseline Race-specific Z-scores Comparing African Americans with White Americans (Reference group).

|  | African American vs White |
| --- | --- |
| Neuropsychological test score | estimate (95% C.I.) |
| MoCA, total score | 0.519*** (0.458, 0.581) |
| Number Span Test Forward, total correct trials | 0.212*** (0.165, 0.258) |
| Number Span Test Forward, longest span | 0.252*** (0.205, 0.298) |
| Number Span Test Backward, total correct trials | 0.273*** (0.228, 0.317) |
| Number Span Test Backward, longest span | 0.183*** (0.139, 0.227) |
| Craft Story 21 Recall Immediate Paraphrase, total units | 0.263*** (0.215, 0.311) |
| Craft Story 21 Recall Delay Paraphrase, total units | 0.301*** (0.250, 0.352) |
| Multilingual Naming Test (MINT), total score | 0.500*** (0.417, 0.582) |
| Animals List Generation, total in 60s | 0.389*** (0.344, 0.433) |
| Vegetables List Generation, total in 60s | 0.151*** (0.108, 0.193) |
| Trail Making Test part A, total correct per minute | 0.139*** (0.097, 0.180) |
| Trail Making Test part B, total correct per minute | 0.170*** (0.128, 0.212) |
| Benson Complex Figure Copy, total score | 0.245*** (0.175, 0.316) |
| Benson Complex Figure Recall, total score | 0.284*** (0.231, 0.336) |

Models adjusted for age, sex, education, baseline comorbidities (diabetes, hypertension, cardiac events), family history, referral source, allowing race-dependent practice effects and different baseline mean and slope across CDR diagnostic groups.

C.I.: Confidence Interval; p-value based on Wald tests: *p<0.05, **p<0.01, ***p<0.001.

Supplementary Table 3. Model-based Estimates of the Difference in Longitudinal Change in Race-specific Z-scores Comparing African Americans with White Americans (Reference group).

|  | Difference in Annualized Change |
| --- | --- |
| Neuropsychological test score | estimate (95% C.I.) |
| MoCA, total score | 0.035 (-0.001, 0.071) |
| Number Span Test Forward, total correct trials | -0.012 (-0.039, 0.014) |
| Number Span Test Forward, longest span | -0.018 (-0.047, 0.012) |
| Number Span Test Backward, total correct trials | 0.007 (-0.021, 0.034) |
| Number Span Test Backward, longest span | 0.019 (-0.011, 0.049) |
| Craft Story 21 Recall Immediate Paraphrase, total units | 0.005 (-0.025, 0.035) |
| Craft Story 21 Recall Delay Paraphrase, total units | 0.013 (-0.016, 0.043) |
| Multilingual Naming Test (MINT), total score | 0.017 (-0.022, 0.056) |
| Animals List Generation, total in 60s | 0.024 (-0.003, 0.050) |
| Vegetables List Generation, total in 60s | 0.005 (-0.022, 0.033) |
| Trail Making Test part A, total correct per minute | -0.003 (-0.029, 0.022) |
| Trail Making Test part B, total correct per minute | 0.001 (-0.024, 0.025) |
| Benson Complex Figure Copy, total score | 0.023 (-0.026, 0.072) |
| Benson Complex Figure Recall, total score | 0.025 (-0.006, 0.055) |

Models adjusted for age, sex, education, baseline comorbidities (diabetes, hypertension, cardiac events), family history, referral source, allowing race-dependent practice effects and different baseline mean and slope across CDR diagnostic groups.

C.I.: Confidence Interval; p-value based on Wald tests: *p<0.05, **p<0.01, ***p<0.001.

Supplementary Table 4. Difference in Baseline Scores Comparing African Americans with White Americans (Reference group): Z-score difference, raw score difference and minimum sample size per arm needed to detect the observed difference in a two-armed clinical trial.

| Neuropsychological test score | Z-score difference | Raw score difference | Minimum sample size per arm |
| --- | --- | --- | --- |
| MoCA, total score | -0.487 | -1.4 | 67 |
| Number Span Test Forward, total correct trials | -0.134 | -0.3 | 875 |
| Number Span Test Forward, longest span | -0.133 | -0.2 | 888 |
| Number Span Test Backward, total correct trials | -0.360 | -0.8 | 122 |
| Number Span Test Backward, longest span | -0.348 | -0.5 | 130 |
| Craft Story 21 Recall Immediate Paraphrase, total units | -0.207 | -0.8 | 367 |
| Craft Story 21 Recall Delay Paraphrase, total units | -0.277 | -1.2 | 205 |
| Multilingual Naming Test (MINT), total score | -0.592 | -1.4 | 45 |
| Animals List Generation, total in 60s | -0.343 | -2.0 | 134 |
| Vegetables List Generation, total in 60s | -0.123 | -0.5 | 1038 |
| Trail Making Test part A, total correct per minute | -0.442 | -8.0 | 81 |
| Trail Making Test part B, total correct per minute | -0.456 | -4.0 | 76 |
| Benson Complex Figure Copy, total score | -0.214 | -1.0 | 343 |
| Benson Complex Figure Recall, total score | -0.097 | -0.4 | 1669 |

Z-score difference is obtained from Model 3, which adjusted for age, sex, education, baseline comorbidities (diabetes, hypertension, cardiac events), family history, referral source, allowing race-dependent practice effects and different baseline mean and slope across CDR diagnostic groups. Raw score difference is back-calculated from Z-score difference by multiplying the standard deviation of each measure obtained from published norms. Minimum sample size per arm is required in a two-arm trial to attain 80% power to detect a signal given by the Z-score difference, using 5% significance tests.

Supplementary Table 5. (a) Baseline Z-score differences between African Americans with White Americans (Reference group). (b) Baseline Z-score differences for between normal control non-progressors and mild cognitively impaired non-progressors. (c) Percentage of observed race difference race compared to the baseline difference between normal control non-progressors and mild cognitively impaired non-progressors.

| Neuropsychological test score | (a) | (b) | (c) |
| --- | --- | --- | --- |
| MoCA, total score | -0.487 | -1.403 | 35% |
| Number Span Test Forward, total correct trials | -0.134 | -0.368 | 36% |
| Number Span Test Forward, longest span | -0.133 | -0.367 | 36% |
| Number Span Test Backward, total correct trials | -0.360 | -0.532 | 68% |
| Number Span Test Backward, longest span | -0.348 | -0.488 | 71% |
| Craft Story 21 Recall Immediate Paraphrase, total units | -0.207 | -1.010 | 21% |
| Craft Story 21 Recall Delay Paraphrase, total units | -0.277 | -1.257 | 22% |
| Multilingual Naming Test (MINT), total score | -0.592 | -0.816 | 33% |
| Animals List Generation, total in 60s | -0.343 | -0.775 | 44% |
| Vegetables List Generation, total in 60s | -0.123 | -0.800 | 15% |
| Trail Making Test part A, total correct per minute | -0.442 | -0.501 | 88% |
| Trail Making Test part B, total correct per minute | -0.456 | -0.617 | 74% |
| Benson Complex Figure Copy, total score | -0.214 | -0.495 | 43% |
| Benson Complex Figure Recall, total score | -0.097 | -1.166 | 8% |

Z-score difference is obtained from Model 3, which adjusted for age, sex, education, baseline comorbidities (diabetes, hypertension, cardiac events), family history, referral source, allowing race-dependent practice effects and different baseline mean and slope across CDR diagnostic groups.

Supplementary Table 6. (a) Baseline Z-score differences between African Americans with White Americans (Reference group). (b) Baseline Z-score differences for three additional years of education. (c) Percentage of observed difference eliminated by a three-year increase in education (capped at 100%)

| Neuropsychological test score | (a) | (b) | (c) |
| --- | --- | --- | --- |
| MoCA, total score | -0.487 | 0.306 | 63% |
| Number Span Test Forward, total correct trials | -0.134 | 0.174 | 100% |
| Number Span Test Forward, longest span | -0.133 | 0.162 | 100% |
| Number Span Test Backward, total correct trials | -0.360 | 0.204 | 57% |
| Number Span Test Backward, longest span | -0.348 | 0.192 | 56% |
| Craft Story 21 Recall Immediate Paraphrase, total units | -0.207 | 0.195 | 94% |
| Craft Story 21 Recall Delay Paraphrase, total units | -0.277 | 0.183 | 67% |
| Multilingual Naming Test (MINT), total score | -0.592 | 0.195 | 33% |
| Animals List Generation, total in 60s | -0.343 | 0.201 | 59% |
| Vegetables List Generation, total in 60s | -0.123 | 0.144 | 100% |
| Trail Making Test part A, total correct per minute | -0.442 | 0.114 | 26% |
| Trail Making Test part B, total correct per minute | -0.456 | 0.192 | 42% |
| Benson Complex Figure Copy, total score | -0.214 | 0.135 | 63% |
| Benson Complex Figure Recall, total score | -0.097 | 0.084 | 86% |

Z-score difference is obtained from Model 3, which adjusted for age, sex, education, baseline comorbidities (diabetes, hypertension, cardiac events), family history, referral source, allowing race-dependent practice effects and different baseline mean and slope across CDR diagnostic groups.
